# Supplementary material for: High Serum Levels of CCL20 Are Associated with Recurrence and Unfavorable Overall Survival in Advanced Melanoma Patients Receiving Immunotherapy
Source: Cancers (Basel). 2024 Apr 29;16(9):1737. doi: 10.3390/cancers16091737 (PMC11083498; doi:10.3390/cancers16091737)
Supplement: Supplementary file 1 [file cancers-16-01737-s001.zip › cancers-2938012-supplementary.docx]

Supplemental Materials

High serum levels of CCL20 are associated with recurrence and unfavorable overall survival in advanced melanoma patients receiving immunotherapy

Julian Kött^1,2*^, Inka Lilott Hoehne^1,2,3*^, Isabel Heidrich^1,2,3^, Noah Zimmermann^1,2^, Kim-Lea Reese^3^, Tim Zell^1,2^, Glenn Geidel^1,2^, Alessandra Rünger^1,2^, Stefan W. Schneider^1,2^, Klaus Pantel^2,3^, Daniel J. Smit ^2,3,#^, Christoffer Gebhardt^1,2,#^

**
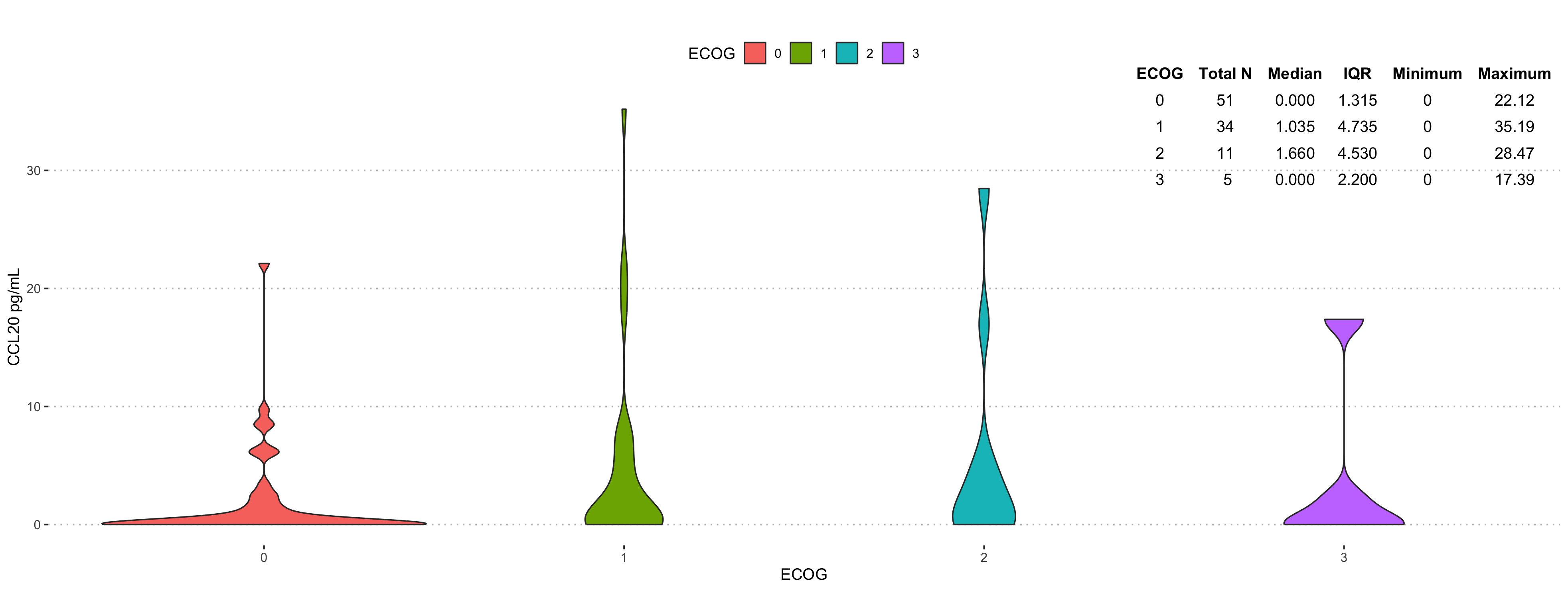
**

**Figure S1: Violin plot of serum CCL20 concentrations grouped by ECOG performance score.** Violin plots of serum CCL20 concentration grouped by ECOG in the advanced melanoma cohort at baseline (n = 101).

**
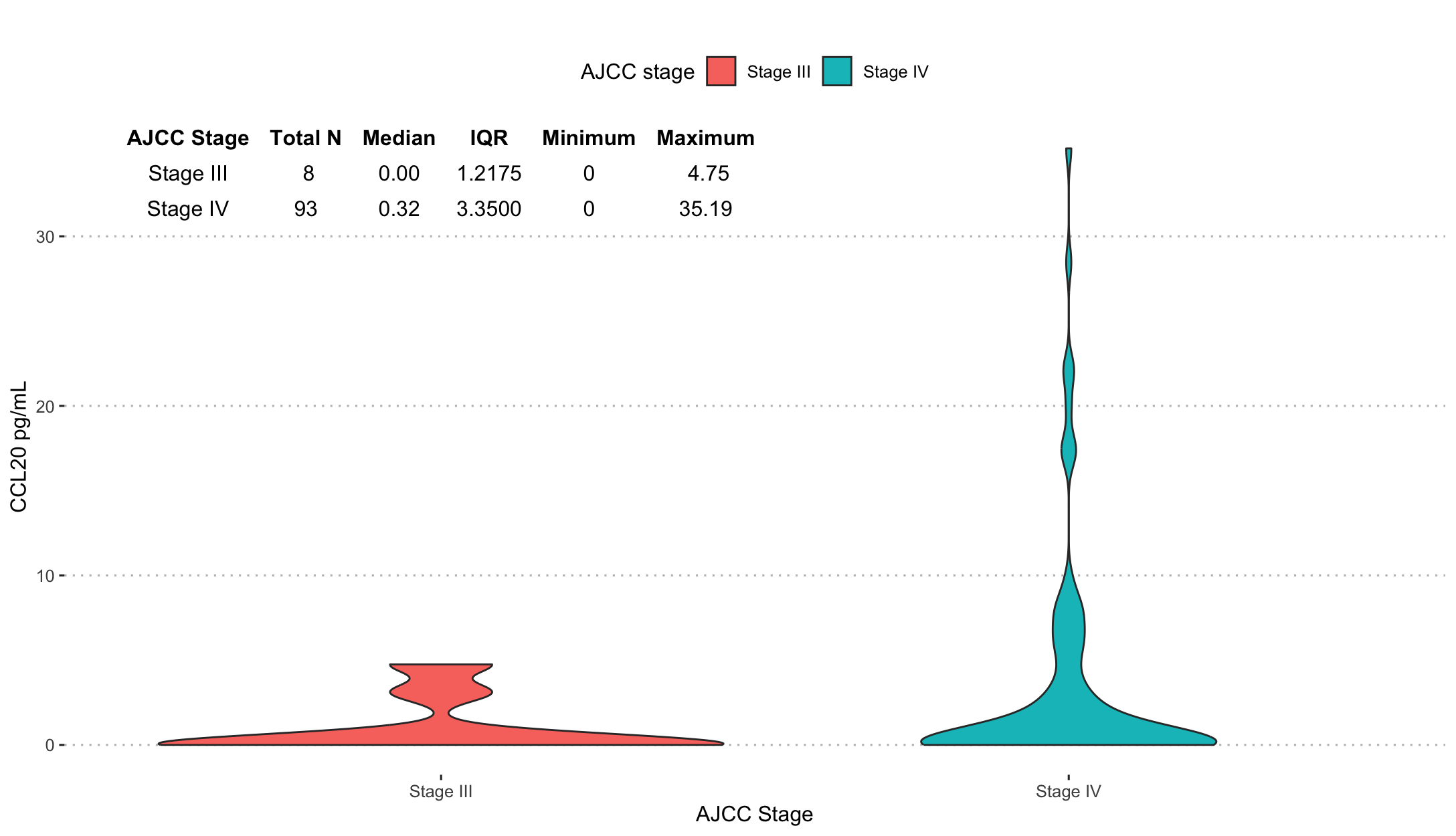
**

**Figure S2: Violin plot of serum CCL20 concentrations grouped by AJCC stage.** Violin plots of serum CCL20 concentration grouped by AJCC stage in the advanced melanoma cohort at baseline (n = 101).
